# Supplementary figures and images for: Analysis of stability law and optimization of slope angle during excavation of deep concave mine slope
Source: PLoS One. 2022 Jul 21;17(7):e0271700. doi: 10.1371/journal.pone.0271700 (PMC9302813; doi:10.1371/journal.pone.0271700)

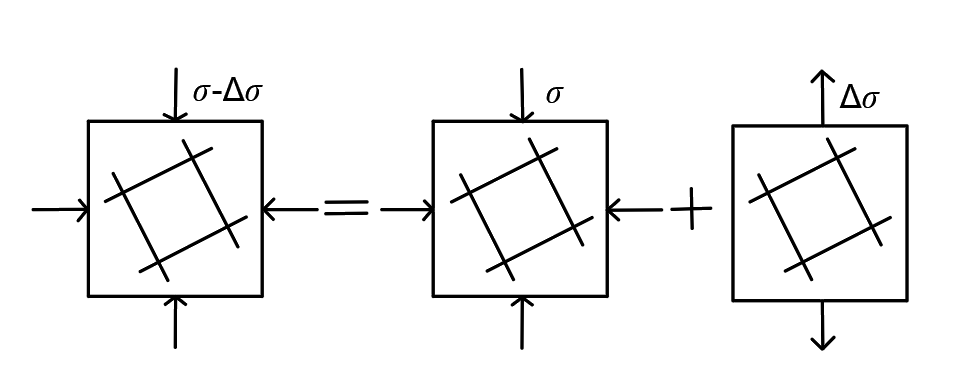

Supplement: S1 Fig — The essence of excavation effect is to remove the load in one or two directions from the slope originally in the three-way stress state and change its original stress state. (TIF) [file pone.0271700.s001.tif]

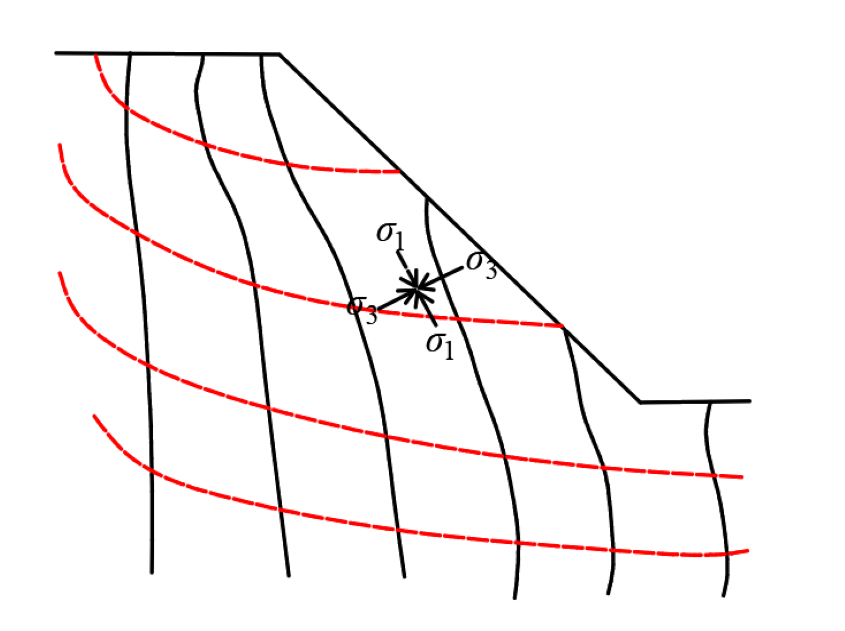

Supplement: S2 Fig — The solid line refers to principal stress trace, red dotted line refers to shear stress trace. The stress variation characteristics in the figure are as follows: (1) As the excavation goes on, the stress changes increase with the decrease of the distance from the excavation surface. (2) The shear stress zone is formed at the foot of slope, which is prone to failure. (TIF) [file pone.0271700.s002.tif]
