# Supplementary material for: Analysis of stability law and optimization of slope angle during excavation of deep concave mine slope
Source: PLoS One. 2022 Jul 21;17(7):e0271700. doi: 10.1371/journal.pone.0271700 (PMC9302813; doi:10.1371/journal.pone.0271700)
Supplement: S1 File — (DOCX) [file pone.0271700.s003.docx]

**Analysis of stability law and optimization of slope angle during excavation of** **deep** **concave mine slope**

Lili Wu^1*^, Keqiang He^2^, Lu Guo^2^, Linna Sun^2^

1. Department of Architectural Engineering, Qingdao Binhai University, Shandong, China.
2. Department of Civil Engineering, Qingdao University of Technology, Shandong, China.

* Corresponding author

E-mail: wll1603863477@163. Com (WLL)

# Midas-GTS finite element model description

The constitutive model of rock and soil mass is Mohr-Coulomb constitutive model, which has simple parameters and high accuracy, and can reflect the characteristics of tribological materials. The Midas-GTS software adopts principle of strength reduction method.

# Principle of strength reduction method

The safety factor of slope stability in strength reduction method is defined as the degree to which the shear strength of rock and soil is reduced when the slope just reaches the critical failure state. Therefore, the safety factor can be defined as the ratio of the actual shear strength of rock and soil mass to the reduced shear strength at critical failure. The main equations involved in strength reduction are:

| $\text{c}^{\text{trial}}\text{=}\text{c}/{\text{F}^{\text{trial}}}$ (1) |
| --- |
| $\text{φ}^{\text{trial}}\text{=}\text{arctan}\left( {\text{tan}\text{φ}}/{\text{F}^{\text{trial}}} \right)$ (2) |

Where $\text{c}^{\text{trial}}$ is the cohesion in the reduction process, *kPa*; $\text{F}^{\text{trial}}$ is the safety factor in the reduction process; *c* is the actual cohesion of the rock and soil mass, *kPa*; $\varphi^{\text{trial}}$ is the angle of internal friction in the reduction process，°; $\text{φ}$ is the actual angle of internal friction of the rock and soil mass，°.
