# Supplementary material for: Analysis of stability law and optimization of slope angle during excavation of deep concave mine slope
Source: PLoS One. 2022 Jul 21;17(7):e0271700. doi: 10.1371/journal.pone.0271700 (PMC9302813; doi:10.1371/journal.pone.0271700)
Supplement: Attachment — Submitted filename: PONE-D-22-08272_reviewer pan.pdf [file pone.0271700.s004.pdf]

PLOS ONE

Analysis of stability law and optimization of slope Angle during excavation of deep concave mine slope

--Manuscript Draft--

**Manuscript Number:**	PONE-D-22-08272
**Article Type:**	Research Article
**Full Title:**	Analysis of stability law and optimization of slope Angle during excavation of deep concave mine slope
**Short Title:**	Analysis of stability law and optimization of slope Angle during excavation of deep concave mine slope
**Corresponding Author:**	lili wu<br>Qingdao University of Technology<br>Qingdao, Shandong Province CHINA
**Keywords:**	Opencast mining；Deep concave slope；Excavation angle；slope stability
**Abstract:**	In order to explore the law of disaster in the process of slope loading and slope Angle optimization, this paper analyzes the sliding force and slope deformation response characteristics during slope excavation, and researches the law of stability change during the process of slope loading. This law provides a theoretical basis for the local or global instability caused by continuous mining and the increase of depression degree of slope and the optimization of slope Angle of excavation. This paper takes Jinchuan mining area in Jinchuan City, Gansu Province, China as an example, selects MIDASGTS finite element software and takes slope angle of each step as simulation parameter to establish a numerical model. Through the simulation of multi-step slope excavation design scheme and four groups of different excavation slope Angle experimental scheme, this paper analyzes the influence of different excavation slope Angle on slope stability under the condition of certain step slope height and width. The results show that with the increase of the slope Angle, the force and deformation of the slope are unfavorable to the slope stability, and the slope stability coefficient decreases gradually. Under the condition of considering stability and maximum recovery, the optimal excavation Angle combination range is determined as 62°~ 65°, 64°~ 67°, 67°~ 69°, 70°~ 71°, 73°, 75°~ 76°, 77°~ 80°. The results verify the loading effect during mine slope excavation, and provide reference for the optimal excavation Angle range of mine slope.
**Order of Authors:**	lili wu
	Keqiang He
	Lu Guo
	Linna Sun
**Additional Information:**	
**Question**	**Response**
**Financial Disclosure**<br><br>Enter a financial disclosure statement that describes the sources of funding for the work included in this submission. Review the submission guidelines for detailed requirements. View published research articles from *PLOS ONE* for specific examples.<br><br>This statement is required for submission and **will appear in the published article** if	Financial Disclosure:<br>This research was supported by the National Natural Science Foundation of China (41372297) (KQ H), the Open Foundation of the Key Laboratory of Geological Hazards on the Three Gorges Reservoir Area (China Three Gorges University) (KQ H), Ministry of Education (2017KDZ03) (KQ H) and the Cooperative Innovation Center of Engineering Construction and Safety in the Shandong Blue Economic Zone. (KQ H)

Powered by Editorial Manager® and ProduXion Manager® from Aries Systems Corporation

the submission is accepted. Please make sure it is accurate.<br><br>**Unfunded studies**<br>Enter: *The author(s) received no specific funding for this work.*<br><br>**Funded studies**<br>Enter a statement with the following details:<br>• Initials of the authors who received each award<br>• Grant numbers awarded to each author<br>• The full name of each funder<br>• URL of each funder website<br>• Did the sponsors or funders play any role in the study design, data collection and analysis, decision to publish, or preparation of the manuscript?<br>• **NO** - Include this sentence at the end of your statement: *The funders had no role in study design, data collection and analysis, decision to publish, or preparation of the manuscript.*<br>• **YES** - Specify the role(s) played.<br><br>* typeset	
**Competing Interests**<br><br>Use the instructions below to enter a competing interest statement for this submission. On behalf of all authors, disclose any competing interests that could be perceived to bias this work—acknowledging all financial support and any other relevant financial or non-financial competing interests.<br><br>This statement is required for submission and **will appear in the published article** if the submission is accepted. Please make sure it is accurate and that any funding sources listed in your Funding Information later in the submission form are also declared in your Financial Disclosure statement.<br><br>View published research articles from *PLOS ONE* for specific examples.	No potential conflict of interest was reported by the authors.

**NO authors have competing interests**<br><br>Enter: *The authors have declared that no competing interests exist.*<br><br>**Authors with competing interests**<br><br>Enter competing interest details beginning with this statement:<br><br>*I have read the journal's policy and the authors of this manuscript have the following competing interests: [insert competing interests here]*<br><br>* typeset	
**Ethics Statement**<br><br>Enter an ethics statement for this submission. This statement is required if the study involved:<br><br>• Human participants<br>• Human specimens or tissue<br>• Vertebrate animals or cephalopods<br>• Vertebrate embryos or tissues<br>• Field research<br><br>Write "N/A" if the submission does not require an ethics statement.<br><br>General guidance is provided below. Consult the submission guidelines for detailed instructions. **Make sure that all information entered here is included in the Methods section of the manuscript.**	N/A

**Format for specific study types**<br><br>**Human Subject Research (involving human participants and/or tissue)**<br>• Give the name of the institutional review board or ethics committee that approved the study<br>• Include the approval number and/or a statement indicating approval of this research<br>• Indicate the form of consent obtained (written/oral) or the reason that consent was not obtained (e.g. the data were analyzed anonymously)<br><br>**Animal Research (involving vertebrate animals, embryos or tissues)**<br>• Provide the name of the Institutional Animal Care and Use Committee (IACUC) or other relevant ethics board that reviewed the study protocol, and indicate whether they approved this research or granted a formal waiver of ethical approval<br>• Include an approval number if one was obtained<br>• If the study involved *non-human primates*, add *additional details* about animal welfare and steps taken to ameliorate suffering<br>• If anesthesia, euthanasia, or any kind of animal sacrifice is part of the study, include briefly which substances and/or methods were applied<br><br>**Field Research**<br><br>Include the following details if this study involves the collection of plant, animal, or other materials from a natural setting:<br>• Field permit number<br>• Name of the institution or relevant body that granted permission	
**Data Availability**<br><br>Authors are required to make all data underlying the findings described fully available, without restriction, and from the time of publication. PLOS allows rare exceptions to address legal and ethical concerns. See the PLOS Data Policy and FAQ for detailed information.	Yes - all data are fully available without restriction

A Data Availability Statement describing where the data can be found is required at submission. Your answers to this question constitute the Data Availability Statement and **will be published in the article**, if accepted.<br><br>**Important:** Stating 'data available on request from the author' is not sufficient. If your data are only available upon request, select 'No' for the first question and explain your exceptional situation in the text box.<br><br>Do the authors confirm that all data underlying the findings described in their manuscript are fully available without restriction?	
**Describe where the data may be found in full sentences. If you are copying our sample text, replace any instances of XXX with the appropriate details.**<br><br>• If the data are **held or will be held in a public repository**, include URLs, accession numbers or DOIs. If this information will only be available after acceptance, indicate this by ticking the box below. For example: *All XXX files are available from the XXX database (accession number(s) XXX, XXX.).*<br>• If the data are all contained **within the manuscript and/or Supporting Information files**, enter the following: *All relevant data are within the manuscript and its Supporting Information files.*<br>• If neither of these applies but you are able to provide **details of access elsewhere**, with or without limitations, please do so. For example:<br><br>*Data cannot be shared publicly because of [XXX]. Data are available from the XXX Institutional Data Access / Ethics Committee (contact via XXX) for researchers who meet the criteria for access to confidential data.*<br><br>*The data underlying the results presented in the study are available from (include the name of the third party*	This information will only be available after acceptance.

and contact information or URL).<br>• This text is appropriate if the data are owned by a third party and authors do not have permission to share the data.<br><br>* typeset	
Additional data availability information:	Tick here if the URLs/accession numbers/DOIs will be available only after acceptance of the manuscript for publication so that we can ensure their inclusion before publication.

Manuscript Click here to access/download;Manuscript;manuscript.docx

Analysis of stability law and optimization of slope Angle during excavation of deep concave mine slope

Lili Wu[1*], Keqiang He[2], Lu Guo[2], Linna Sun[2]

1. Department of Architectural Engineering, Qingdao Binhai University, Shandong, China.
2. Department of Civil Engineering, Qingdao University of Technology, Shandong, China.

* wll1603863477@163. Com (LLW)

Abstract

In order to explore the law of disaster in the process of slope loading and slope Angle optimization, this paper analyzes the sliding force and slope deformation response characteristics during slope excavation, and researches the law of stability change during the process of slope loading. This law provides a theoretical basis for the local or global instability caused by continuous mining and the increase of depression degree of slope and the optimization of slope Angle of excavation. This paper takes Jinchuan mining area in Jinchuan City, Gansu Province, China as an example, selects MIDASGTS finite element software and takes slope angle of each step as simulation parameter to establish a numerical model. Through the simulation of multi-step slope excavation design scheme and four groups of different excavation slope Angle experimental scheme, this paper analyzes the influence of different excavation slope Angle on slope stability under the condition of certain step slope height and width. The

results show that with the increase of the slope Angle, the force and deformation of the slope are unfavorable to the slope stability, and the slope stability coefficient decreases gradually. Under the condition of considering stability and maximum recovery, the optimal excavation Angle combination range is determined as 62°~ 65°, 64°~ 67°, 67°~ 69°, 70°~ 71°, 73°, 75°~ 76°, 77°~ 80°. The results verify the loading effect during mine slope excavation, and provide reference for the optimal excavation Angle range of mine slope.

Introduction

In mining engineering, open-pit mining is a common form of mining. Studying the stability change law of this kind of slope and analyzing the excavation angle in the mining process are two important contents in the field of mine slope. Weibei area is the largest limestone resource area in Shaanxi Province in China, and the complex geological environment and harsh environment make the mining area have serious geological disaster hidden danger. There is a sliding trend in both sides during excavation, and the maximum vertical displacement is 2.1 cm during the excavation process [1]. In Xincheng gold mine project in China, due to the excavation of the mine, the rock mass in the mine has obvious displacement, which has a serious impact on the safety of the mining process [2]. In addition, water outbursts and rock bursts often occur

during the mining of coal seams under water-rich sandstone strata with thicknesses exceeding 50 m, otherwise called ultrathick-and-hard strata (UTHS), which are common throughout the mining areas of northwestern China [3]. The Higashi-Shikagoe limestone quarry is an open-pit mine situated in Hokkaido Prefecture, Japan, that has experienced four slope failure incidents since 1996 [4]. In excavation engineering, by analyzing the stability of a group of headings driven in high horizontal stress fields in the copper ore mines of the Legnica-Glogow Belt (LGCB), the direction of driving the headings in the field of increased horizontal stresses may be of key importance for the stability of the headings in LGOM mines [5-7]. As a high-efficiency and low-consumption mining mode, multi-middle section combined backfilling mining (MMSCBM) is becoming more and more widely used in metal mines. MMSCBM can effectively buffer the excavation's disturbance to the surrounding rock, adjust the stress release mode, change the stress concentration area, and improve the stability [8]. From the perspective of geotechnical engineering, rock pillars can be defined as the in-situ rock between two or more underground openings. However, in the analysis of hard rock pillars and indirectly in synthetic rock mass models to determine rock mass strength, it is very important to consider the actual stress level and stress path imposed on the pillars due to the excavation sequence of pillars [9]. From the perspective of excavation

stability, the complex nonlinear mechanical behavior of the combined system of rock and coal surrounding mine openings is a major obstacle for excavation, stability, and support design in mining thin coal seams [10-15]. The geological structure of the Changshanhao open-pit mine in Urad Middle Banner, Inner Mongolia, China is extremely complicated, and slope instability has frequently occurred in various forms, such as wedge sliding, bedding sliding, and toppling failure. Slope failure is the consequence of endogenic and exogenic integration, including physical and mechanical properties of the rock mass, geological structures such as faults and joints, and human-caused factors such as blasting and excavation disturbances [16]. In underground mining activities, rainfall and the dip of an excavated rock slope also has a potentially important impact on the initiation and reactivation of the slope deformation, especially on the steep rock slope [17-19]. The above studies have routinely analyzed the stability law of mine slope, but the dynamic change mechanism of excavation instability of this kind of slope is less. Taking the large quartz open-pit mine of Jinchuan Group in Gansu Province in China as an example, this study analyzes the influence of different excavation angles on slope stability, in order to obtain the optimal combination range of excavation angle parameters and provide a certain reference for the construction of related projects.

Study on the influence law of excavation loading process on slope stability in deep concave mine

Evolution characteristics of slope sliding dynamics and deformation response in deep concave mines

In the process of excavation of slope in deep pit mine, the initial stress equilibrium state in slope body is affected by the excavation behavior and the stress redistribution occurs [20-23]. In fact, the fundamental impact of excavation behavior is to increase the sliding power of the slope, which is equivalent to continuously loading the slope during the whole construction process. At the same time, there will be corresponding displacement near the slope face with the progress of excavation [24-26]. It is assumed that the slope of deep concave mine is homogeneous, and the homogeneous body is isotropic, and the thickness of the slope changes uniformly. Taking the potential sliding strip of slope as the research object, the stress of sliding strip under the dynamic action of excavation is as follows (Fig 1).

Where H_{i1} and H_{i1} are respectively the height before and after the excavation of the sliding strip, *m*; G_i and G_i’ are respectively the gravity before and after the excavation of the sliding strip, *KN* ;ΔP_i and ΔP_i’ are respectively the remaining sliding forces before and after the excavation of the sliding strip, *KN*; R_i is the anti-sliding force of

the sliding strip, *KN*; N_i is the effective stress of the base, *KN*; $h^{'}$ is the increased height of the sliding strip after excavation, *m*; *h* is the excavation height of sliding strip, *m*; θ is the included Angle between the sliding surface of the sliding strip and the horizontal plane.

Fig 1 shows that the sliding force and anti-sliding force are the gravity components of the slide block. The sliding force is represented by T, and the anti-sliding force is represented by R. The sliding force and anti-sliding force of the sliding strip before and after excavation are shown as follows:

Sliding force before excavation：

$$T_1 = G_1 \sin\theta = (\gamma H_1)\sin\theta \tag{1}$$

Sliding force after excavation：

$$T_2 = G_2 \sin\theta = (\gamma H_2)\sin\theta \tag{2}$$

Anti-sliding force before excavation：

$$R_1 = G_1 \cos\theta \tan\varphi + cl = (\gamma H_1)\cos\theta \tan\varphi + cl \tag{3}$$

Anti-sliding force after excavation：

$$R_2 = G_2 \cos\theta \tan\varphi + cl = (\gamma H_2)\cos\theta \tan\varphi + cl \tag{4}$$

Where T_1 is sliding force before excavation of sliding strip, *KN*; T_2 is sliding force after excavation of sliding strip, *KN*; R_1 is the anti-sliding force of sliding strip before

excavation, *KN*; R_2 is the anti-sliding force of the sliding strip after excavation, *KN*; G_1 is the gravity before excavation of sliding strip, *KN*; G_2 is the gravity after excavation of sliding strip, *KN*; *C* is cohesion, *KPa*; φ is the Angle of internal friction，°; θ is the included Angle of the sliding surface, ° ; l is the bottom width of the slider block，*m*; γ is the natural gravity of rock and soil mass，KN/m^3. Other symbols have the same meaning as above.

Therefore, the increment of sliding power of sliding strip caused by excavation is:

$$\Delta P = P_2 - P_1 = (T_2 - T_1) - (R_2 - R_1) = (H_2 - H_1)(\gamma \sin\theta - \gamma \cos\theta \tan\varphi) \tag{5}$$

Where P_1 is the remaining sliding force before the excavation of sliding strip, *KN*; P_2 is the remaining sliding force after excavation of sliding strip，*KN* ; ΔP is the sliding power increment of the sliding strip, *KN*; Other symbols have the same meaning as above.

Formula 5 shows that for specific slope, $(\gamma \sin\theta - \gamma \cos\theta \tan\varphi)$ is the constant value, and $(H_2 - H_1)$ is the increase in sliding strip height caused by excavation. Only when the slope is excavated downward, the volume of potential sliding soil will increase, and the height of sliding strip will increase. Therefore, it can be considered that the excavation depth corresponds to the remaining sliding force increment in the process of slope excavation in deep depression mine.

In the calculation of the slope body, the slope body is assumed to be ideal elastic-plastic, and the stress and deformation physical parameters of the slope body section are averaged [27]. According to the basic principle of elastic-plastic mechanics, the relationship between the sliding force and displacement variation caused by the excavation and loading of slope in deep concave mine is expressed as follows. :

$$\Delta S_i = l\bar{\varepsilon}_i \tag{6}$$

$$\bar{\varepsilon}_i = \frac{\bar{\sigma}_i}{\bar{E}_i} \tag{7}$$

$$\bar{\sigma}_i = \frac{\Delta P_i}{V_i} \tag{8}$$

Where ΔS_i、$\bar{\varepsilon}_i$ and $\bar{\sigma}_i$ are the displacement variation, mean value of strain and mean value of stress of sliding strip caused by excavation loading; $\bar{E}_i$ is the mean value of deformation modulus of sliding strip; V_i is the volume of sliding strip (unit width condition), $V_i = H_i l \cos\theta$。

Formula 5 to Formula 8 show that: with other factors relatively unchanged, the slope sliding dynamic change and displacement change caused by slope excavation in deep and concave mines mainly depend on the change of excavation height. With the increase of excavation height, the sliding force and displacement change of slope increase. Therefore, it can be considered that the excavation loading process is the main dynamic factor affecting the sliding dynamic change and displacement change of mine slope.

Stability evolution of mine slope under excavation loading condition

According to the above formula and the limit equilibrium principle of slope stability, the stability coefficient of mine slope after excavation is determined as follows:

$$F_{si}=\frac{(\gamma H_{i2})cos\theta tan\varphi+cl}{(\gamma H_{i2})sin\theta}=\frac{cos\theta tan\varphi}{sin\theta}+\frac{cl}{\gamma H_{i2}sin\theta} \quad (9)$$

Formula 9 shows that only the excavation height variation is the variation parameter in the process of mining slope loading increase, while other physical and geometric parameters are relatively constant. Therefore, the slope stability coefficient is directly related to the dynamic change process of excavation loading.

Study Area

Landslide situation

Jinchuan Group quartz open-pit mine is located in Jinchuan District, Jinchang City, Gansu Province, China (Fig 2), and began mining in 1980. The ore area is cut by a series of Sw-Ne strike thrust faults (Fig 3), and the intrusive ore bodies are divided into several sections, which are divided into I, Ⅱ, Ⅲ and Ⅳ mining areas by Gansu Geological Survey Institute [28]. Among them, mine I and Ⅲ are mined and managed by Longshou Mine of Jinchuan Company, mine Ⅱ is mined and managed by West No 2 Mine and mine Ⅳ is mined and managed by East No 3 Mine [29]. The mining distribution of Jinshan mine is as follows. (Fig 4). Up to now, the mining area has

formed a total of 10 steps of 1856m, 1846m, 1836m, 1826m, 1816m, 1792m, 1780m, 1768m, 1756m, 1744m and 1732m from top to bottom (Fig 5). With the development of mining, there have been many failures of the slope, such as local landslides on platforms 1816 and 1768 (Fig 6).

Numerical simulation

According to the actual situation of the mining area, the section 5C~1C of the north slope of the mining area where local landslide occurred was selected as the modeling object, including 7 steps (between 1826m and 1732m) (Fig 7).

According to the geological survey and test data provided by the mining area, the physical and mechanical parameters of the rock mass in the mining area are as follows (Table 1) [30].

Table 1. Rock and soil parameters of slope. [30]

**Stratum**	**Thickness （m）**	***E* （MPa）**	***c*（KPa）**	**Φ（°）**	**γ（KN/m³）**	**μ**
**Quaternary**	15	1038	26.0	26.00	16.5	0.15

**Carboniferous-Permian System**	22	1520	109.4	29.75	26.2	0.14
**Upper formation of Lower Paleozoic**	30	1392	245.1	33.00	27.8	0.21
**Lower Paleozoic lower group**	/	1798	148.2	35.00	27.0	0.24

According to the selected research object, four kinds of excavation slope Angle parameters are set in the experimental scheme. The excavation Angle increases gradually in schemes 1 to 2, and the overall steepening of multi-step slope in schemes 3 to 4 (Table 2). Before the numerical simulation of the experimental scheme, the design scheme of the mine slope is simulated first, which is convenient for comparative analysis.

Table 2. The parameters of excavation slope Angle in the experimental scheme (°).

**Schemes**	**1826-1816**	**1816-1792**	**1792-1780**	**1780-1768**	**1768-1756**	**1756-1744**	**1744-1732**

**1**	60	62	65	68	71	74	77
**2**	62	64	67	70	73	76	80
**3**	65	67	69	71	73	75	77
**4**	66	68	70	72	74	76	78

Midas~GTS software was used to calculate the overall stability coefficient of slope respectively for the design scheme and the experimental scheme. According to the calculation results, the stress change, displacement change, plastic zone distribution and overall stability coefficient after excavation were compared and analyzed. By referring to the relevant specifications, the limit value of slope stability coefficient is determined as 1.3, and the stability coefficient of each scheme was compared with the specification value.

Results analysis

Analysis of design scheme

Stress field analysis

The stress cloud diagram during slope excavation is shown below. Before the excavation, the horizontal stress of the slope is all compressive stress, and the stress field is uniform strip distribution. When the slope is excavated to 1732 platform, the

horizontal stress of the slope body is compressive stress, which increases with the increase of slope depth. Stress concentration occurs at the foot of each step slope, and the stress in other parts is basically distributed in a uniform band (Fig 8). Similar to the distribution of horizontal stress field, the excavation also destroys the vertical initial stress state of the slope and causes the stress redistribution of the slope. The vertical stress of the slope is also compressive stress, but there is no stress concentration. (Fig 9). Therefore, the vertical stress has little damage to the slope.

Considering that the horizontal stress has a great influence on the slope, the element located in the middle of each step and the top of the slope is selected in the model, and its horizontal stress value is extracted. Thus, the curve of horizontal stress variation of each step during slope excavation is drawn（Fig 10）. It can be seen from the figure that with the progress of excavation, the horizontal stress of the slope and the top has the same change trend, and the horizontal compressive stress of the lower step gradually decreases, with a decrease of about 50%. At the same time, the horizontal compressive stress of the first and second steps is also in a stable state without increasing trend. In addition, for the same step, the value of horizontal compressive stress at the top of the slope is less than that at the middle of the slope, which is caused by the fact that tensile failure usually appears at the top of the slope first. In addition, because each step is in

the "underground" state first, and then excavated to form a part of the slope. Therefore, the change in the early stage of the curve in Figure 8 shows an irregular "rise and fall" phenomenon, and the change trend is consistent after excavation.

Displacement field analysis

Fig 11 is the cloud map of horizontal and vertical displacement during slope excavation. With the increase of excavation depth, the direction of horizontal displacement near the slope face changes gradually from deviating to facing the face, and the value of horizontal displacement increases with the increase of slope depth. The vertical displacement during excavation is shown in the figure. For each step excavated, obvious unloading rebound occurs on the step and at the bottom of the pit [31, 32].

The element located in the middle of each step is selected in the model, and the horizontal displacement value is extracted to make the curve of the horizontal displacement of each step in the process of slope excavation (Fig 12). As can be seen from the figure, the step at the upper part of the slope has a negative displacement, and the displacement gradually increases and tends to be stable. The step at the lower part of the slope has a positive displacement, and the displacement value increases gradually. If the excavation continues downward, it can be predicted that when the negative displacement of the upper step of the slope tends to be stable, its value will gradually

develop to the positive displacement, while the positive displacement of the lower step will continue to increase.

Plastic zone and the global stability analysis

According to the distribution range of plastic zones, plastic zones are generated from the foot of each step slope, and then start to develop upward, and gradually connect and penetrate (Fig 13). The stability coefficient of slope during excavation can be obtained by strength reduction method (Fig 14). Fig 14 shows that the stability coefficient decreases with the excavation of the slope. At the fourth step of excavation, the stability coefficient rises, which is caused by the redistribution of the slope stress during the dynamic excavation. The cracks or shear failure caused by the previous excavation will close with the redistribution of stress, resulting in the new stability of rock and soil weight；It can also be seen from the cloud map of plastic zone that during the fourth excavation step, the range of plastic zone generated by the third and fourth steps is very small compared with other parts. This also indicates that when the slope is excavated to the fourth layer, its stability coefficient is larger than that of the first three steps. But in general, with the progress of excavation, the stability of slope gradually decreases until the end of mining.

The element located in the middle and foot of each step is selected in the model, and

its maximum shear stress value is extracted, and the maximum shear stress curve of each step in the process of slope excavation is made. Fig15 shows that with the progress of excavation, the variation trend of maximum shear stress at the middle and foot of slope is basically the same. The shear stress of the first and second steps is relatively stable, while the shear stress of the other steps decreases before excavation. However, after excavation, the shear stress begins to increase and gradually tends to be stable. In addition, for the same step, the shear stress at the foot of the slope is greater than that at the middle of the slope, which is caused by the shear failure usually appearing at the foot of the slope first. It is worth noting that the shear stress curves in the early stage all show a downward trend, which is due to the fact that each step is "underground". However, after the excavation, the shear stress variation trend of each step is basically the same.

Analysis of experimental scheme

According to the simulation results of schemes 1~2, the distribution rules of horizontal stress, horizontal displacement and vertical stress and vertical displacement are basically similar to those of the design scheme. Among them, the horizontal displacement in schemes 1 to 2 changes with the change of slope Angle, the negative horizontal displacement gradually decreases, and the positive horizontal displacement

gradually increases (Tables 3 and 4). This range gradually expands from the bottom of the slope to the top of the slope. The vertical displacement decreases gradually with the increase of slope Angle, that is, the recovery value of excavation unloading of rock mass decreases, but the reduction range is limited.

Table 3. Calculation of horizontal displacement of 1780-1768m stair under different excavation conditions.

Excavation steps(m)	Horizontal displacement (m)				Variation value of horizontal displacement (m)		
	Scheme 1	Scheme 2	Scheme 3	Scheme 4	Scheme 1-2	Scheme 2-3	Scheme 3-4
**1826-1816**	0.0007	0.0007	0.0007	0.0007	0	0	0
**1816-1792**	0.0017	0.0015	0.0016	0.0016	-0.0002	0.0001	0
**1792-1780**	0.0008	0.0004	0.0006	0.0006	-0.0004	0.0002	0
**1780-1768**	-0.0001	-0.0004	-0.0001	0.0000	-0.0003	0.0003	0.0001
**1768-1756**	-0.0011	-0.0013	-0.0008	-0.0006	-0.0002	0.0005	0.0001
**1756-1744**	-0.0013	-0.0014	-0.0007	-0.0002	-0.0001	0.0007	0.0005
**1744-1732**	-0.0008	-0.0009	0.0001	0.0009	-0.0001	0.001	0.0008

Table 4. Calculation of horizontal displacement of 1756-1744m stair under different excavation conditions.

Excavation steps(m)	Horizontal displacement (m)				Variation value of horizontal displacement (m)		
	Scheme 1	Scheme 2	Scheme 3	Scheme 4	Scheme 1-2	Scheme 2-3	Scheme 3-4
**1826-1816**	0.0010	0.0011	0.0011	0.0011	0.0001	0	0
**1816-1792**	0.0039	0.0040	0.0041	0.0041	0.0001	0.0001	0
**1792-1780**	0.0051	0.0053	0.0054	0.0053	0.0002	0.0001	-0.0001
**1780-1768**	0.0057	0.0061	0.0061	0.0060	0.0004	0	-0.0001
**1768-1756**	0.0055	0.0060	0.0060	0.0055	0.0005	0	-0.0005
**1756-1744**	0.0091	0.0096	0.0109	0.0131	0.0005	0.0013	0.0022
**1744-1732**	0.0114	0.0125	0.0151	0.0183	0.0011	0.0026	0.0032

The distribution of plastic zone in schemes 1~2 is different from that in the design scheme: with the increase of slope Angle, the plastic zone at the foot of the slope of steps 3~4 is gradually reduced compared with that in the design scheme, while the plastic zone at steps 5~7 is gradually increased compared with that in the design scheme. Therefore, there is a greater risk of local landslide at steps 5 to 7, which needs to be

controlled during construction (Fig 16).

According to the simulation results of schemes 3-4, the distribution rules of horizontal displacement, vertical displacement and vertical stress of slope are consistent with those of the schemes mentioned above. However, the distribution of horizontal tensile stress in schemes 3-4 gradually moves to the upper part of the slope. With the change of slope Angle in scheme 3 and 4, the plastic zone at the foot of slope of step 3 and 4 continues to decrease, and the plastic zone at the foot of slope of step 5 to 7 continues to expand to the top of slope, while the plastic zone at the foot of slope of step 1 and 2 is gradually connected.

Table 5. Stability coefficients of schemes 1 to 4.

**The excavation platform**	**Scheme 1**	**Scheme 2**	**Scheme 3**	**Scheme 4**
**1826-1816**	2.0816	2.1094	2.0500	2.0313
**1816-1792**	1.5781	1.5891	1.5156	1.5262
**1792-1780**	1.5813	1.5563	1.5039	1.5031
**1780-1768**	1.6004	1.5563	1.5531	1.4656
**1768-1756**	1.5688	1.5441	1.5156	1.4750

**1756-1744**	1.4820	1.4006	1.3953	1.3898
**1744-1732**	1.3719	1.3570	1.3313	1.2875

As shown in Table 5 and Fig 17, the slope stability coefficient gradually decreases with the increase of slope excavation depth and slope Angle. When the first five steps are excavated, the stability coefficients of schemes 1 to 2 are all greater than 1.3, and the reduction of stability coefficients of the last two steps is also limited after the excavation is completed. Considering the control of the maximum mining amount and the calculation results of the strength reduction method in Midas GTS finite element software tend to be safe, the slope Angle of scheme 2 can be considered as the limit range temporarily. With the increase of the overall slope Angle in scheme 3 and 4, the stability coefficient also shows a trend of gradual decrease. However, the final safety coefficient of scheme 3 and 4 decreases to around 1.3, and the stability coefficient of scheme 4 is lower than 1.3, so it is not considered. Considering that the stability coefficient obtained by Midas GTS finite element software tends to be safe and combined with the above mentioned, the combination range of the optimal slope step slope Angle is finally determined to be between scheme 2 and scheme 3.

Conclusions

Under the condition of excavation loading, this paper analyzes the sliding force,

deformation response and stability change of slope in deep pit mine, carries out numerical simulation of slope mining in open pit deep pit mine in Jinchang, Gansu Province, China, and draws the following conclusions:

（1）The excavation loading process of mine slope is a one-way process of reducing stability. The loading effect of this kind of slope is shown as the increase of sliding power and deformation response. The variation of sliding force is mainly affected by the excavation height, slope inclination Angle and physical and mechanical properties of slope body. Among them, the change of excavation height is the main dynamic factor leading to the increase of sliding force of mine slope, which indicates that the process of excavation loading is a subjective factor affecting the stability of this kind of slope.

（2）With the progressive mining of open-pit multi-stage slope, both the horizontal stress and the vertical stress increase gradually, and the stress concentration at the foot of slope mainly depends on the horizontal stress; The horizontal displacement gradually changed from negative to positive, and the uplift of slope and pit bottom also increased gradually; The plastic zone appeared at the foot of step slope and gradually expanded upward. The overall stability coefficient of slope shows a downward trend.

（3）With the increase of slope Angle, the stability coefficient of slope decreases gradually under the four experimental schemes. A small recovery in the safety factor in

individual scenarios is caused by stress redistribution and the re-closure of existing fractures or shear bands.

（4）According to the results of design scheme and experiment scheme, the optimal value range of slope Angle of each step is finally determined between scheme 2 and Scheme 3. That is 62°~ 65°、64°~ 67°、67°~ 69°、70°~ 71°、73°、75°~ 76°、77°~ 80°.

Acknowledgments

The authors are grateful to anonymous reviewers for their valuable suggestions.

Author Contributions

Conceptualization: Lili Wu, Keqiang He.

Data curation: Lili Wu, Lu Guo, Linna Sun.

Funding acquisition: Lili Wu.

Methodology: Lili Wu, Keqiang He.

References

1. Liu KM, Li H, Pang SH, Mi M, Chen JP, Sun K et al. Numerical simulation analysis of slope instability and failure of limestone mine in Weibei. Advances in Civil Engineering. 2021. https:// doi.org/10.1155/2021/5991348
2. Yang Y, Zhang MS, Hu GJ, Guan K. Simulations of goaf surface subsidence via filling control. PLOS one. 2022; 17(1). https:// doi.org/10.1371/JOURNAL.PONE.0261740
3. Wang XZ, Zhu WB, Xie JL, Han HK, Xu JM, Tang ZY, et al. Borehole-Based monitoring of

mining-induced movement in ultrathick-and-hard sandstone strata of the Luohe formation. Minerals. 2021; 11(11): 1157-1157. https:// doi.org/10.3390/MIN11111157

4. Amagu CA, Zhang C, Kodama JI, Shioya K, Yamaguchi T, Sainoki A, et al. Displacement measurements and numerical analysis of Long-Term rock slope deformation at Higashi-Shikagoe limestone quarry, Japan. Advances in Civil Engineering. 2021; 2021. https:// doi.org/10.1155/2021/1316402
5. AdachPawelus K, Pawelus D. Influence of driving direction on the stability of a group of headings located in a field of high horizontal stresses in the polish underground copper mines. Energies. 2021; 14(18): 5955-5955. https:// doi.org/10.3390/EN14185955
6. Guan XM, Wang XC, Nie KQ, Zhou HZ, Yu YL. Stability analysis and parameters optimization of an Open-pit Slope. Safety in Coal Mines. 2018; 49(05):71-74. https:// doi.org/10 .13347/j cnki mkaq .2018 05 018.
7. Gao XC. Experiment research on stability and dynamaic displacement characters due to slope excavation in Open-pit mine. Xi'An University of Science and Technology. 2006.
8. Zhu HX, Han LJ, Meng QB, Li XQ, Tian ML, Meng LD. Study on surrounding rock disturbance effect of multi-middle section combined backfilling mining in inclined iron ore. Arabian Journal of Geosciences. 2021; 14(18). https:// doi.org/10.1007/S12517-021-07969-5
9. Elmo D, Cammarata G, Brasile S. The role of numerical analysis in the study of the behaviour of hard-rock pillars. IOP Conference Series: Earth and Environmental Science. 2021; 833(1). https:// doi.org/10.1088/1755-1315/833/1/012132
10. Zhao Rui. Analysis for bearing performance and construction mechanical behavior of supporting structure for the large cross-section tunnel by half bench CD method. PlOS one. 2021; 16(8). https:// doi.org/10.1371/JOURNAL.PONE.0255511
11. Yu WJ, Wu GS, Pan B, Wu QH, Liao Z. Experimental investigation of the mechanical properties of sandstone coal bolt specimens with different angles under conventional triaxial compression. international journal of geomechanics. 2021; 21(6). https:// doi.org/10.1061/(ASCE)GM.1943-5622.0002005
12. Shannon M G. Reliability assessment of an open pit slope on bong iron ore mine. Journal of Geoscience and Environment Protection. 2021; 09(03):31-43. https:// doi.org/10.4236/GEP.2021.93003
13. Li XS, Wang YM, Liu ZF, Zhao K. Catastrophic mechanism analysis of deformation and instability of the layered rock slope. IOP Conference Series: Earth and Environmental Science. 2021; 634(1):012031-. https:// doi.org/10.1088/1755-1315/634/1/012031
14. Satyanarayana I, Budi G, Murmu S. Stability analysis of a deep highwall slope using numerical modelling and statistical approach—a case study. Arabian Journal of Geosciences. 2021; 14(3). https:// doi.org/10.1007/S12517-021-06476-X

15. Ghazdali O, Tagma T, Rziki S, Moustadraf J. Application of empirical analysis to underground mine a study case In Morocco. Journal of Physics: Conference Series. 2021; 1743(1): 012031-. https:// doi.org/10.1088/1742-6596/1743/1/012031
16. Ren SL, Tao ZG, He MC, Pang SH, Li MN, Xu HT. Stability analysis of open-pit gold mine slopes and optimization of mining scheme in Inner Mongolia, China. Journal of Mountain Science. 2020; 17(12):2997-3011. https:// doi.org/10.1007/S11629-020-6217-X
17. Li ZQ, Xue YG, Li SC, Zhang LM, Wang D, Li Bin, et al. Deformation features and failure mechanism of steep rock slope under the mining activities and rainfall. Journal of Mountain Science. 2017; 14(1):31-45. https:// doi.org/10.1007/s11629-015-3781-6
18. Yilmaz M, Ertin A, Er S, Tugrul A. Numerical modelling of steep slopes in open rock quarries. Journal of the Geological Society of India. 2018; 91(2):232-238. https:// doi.org/10.1007/s12594-018-0841-x
19. Rasheed A, Muili A, Arum C. Open pit slope design of ajabanoko iron ore deposit, kogi state, nigeria. journal of mining world express. 2014; 3:25-32. https:// doi.org/10.14355/mwe.2014.0302.01
20. Zhu C, He MC, Murat K, Cui XB, Tao ZG. Investigating toppling failure mechanism of anti-dip layered slope due to excavation by physical modelling. Rock Mechanics and Rock Engineering. 2020; 53 (prepublish):1-22. https:// doi.org/10.1007/s00603-020-02207-y
21. Tao ZG, Zhu C, He MC, Liu KM. Research on the safe mining depth of anti-dip bedding slope in Changshanhao Mine. Geomechanics and Geophysics for Geo - Energy and Geo – Resources. 2020; 6(4):595-606. https:// doi.org/10.1007/s40948-020-00159-9
22. Alison M, Ismet C, Joung O. Methods applied in Australian industry to evaluate coal mine slope stability. International Journal of Mining Science and Technology. 2020; 30(2):151-155. https:// doi.org/10.1016/j.ijmst.2019.11.001
23. Boonchai U, Rithy O, Thirapong P, Mohammad HK. Investigation of stability and failure mechanism of undercut slopes by three-dimensional finite element analysis. KSCE Journal of Civil Engineering. 2018; 22(5):1730-1741. https:// doi.org/10.1007/s12205-017-2011-x
24. Nie L, Li ZC, Zhang M, Xu LN. Deformation characteristics and mechanism of the landslide in West Open-Pit Mine, Fushun, China. Arabian Journal of Geosciences. 2015; 8(7):4457-4468. https:// doi.org/10.1007/s12517-014-1560-2
25. Feng SJ, Zhao XF, Sun SG. Research on the slope sliding mechanism of excavation of steeply inclined mine from open-pit to underground. Advanced Materials Research. 2014; 3246(962-965):1075-1078. https:// doi.org/1075-1078. 10.4028/www.scientific.net/AMR.962-965.1075
26. Chang LS, Li X, Zhang RP, Chen YN. Analysis on flac large deformation mode of rock mass due to transform from Open-Pit to underground mining. Applied Mechanics and Materials. 2013; 2545(353-356):957-960. https:// doi.org/10.4028/www.scientific.net/AMM.353-

356.957

27. He KQ, Guo L. Research on the prediction parameter and evaluation method of displacement coupled with water dynamics of the reservoir landslides. Journal of Hydraulic Engineering. 2017; 48(5): 516-525. https:// doi.org/10.13243/j.cnki.slxb.20160881
28. Prichard HM, Knight RD, Fisher PC, Iain M, Zhou MF, Christina YW. Distribution of platinum-group elements in magmatic and altered ores in the Jinchuan intrusion, China: An example of selenium remobilization by post magmatic fluids. Mineralium Deposita. 2013; 48(6):767-786.
29. Geng XK. Study of the subsidence monitoring in dragon head mine area with series InSAR technology. Shandong University of Science and Technology. 2018. https:// doi.org/10.27275/d.cnki.gsdku.2018.000398
30. Zhang YJ, Chen GP, Sa ZY, Yang DF, Li QL, Li B. Study on slope shape effect of multistage slope blasting vibration in rock mass. Safety in Coal Mines. 2015; 46(09):71-74. https:// doi.org/10.13347/j.cnki.mkaq.2015.09.020
31. Zhong FS. The rock slope stability assessment and dynamic analysis at cape lambert Open-pit. Central south university. 2012.
32. Ma XY. Study on the excavation unloding dynamic effect and stability evolution rule of the open-pits rock slope. Qingdao Technological University. 2014.

Click here to access/download;Figure;Figure 1.docx

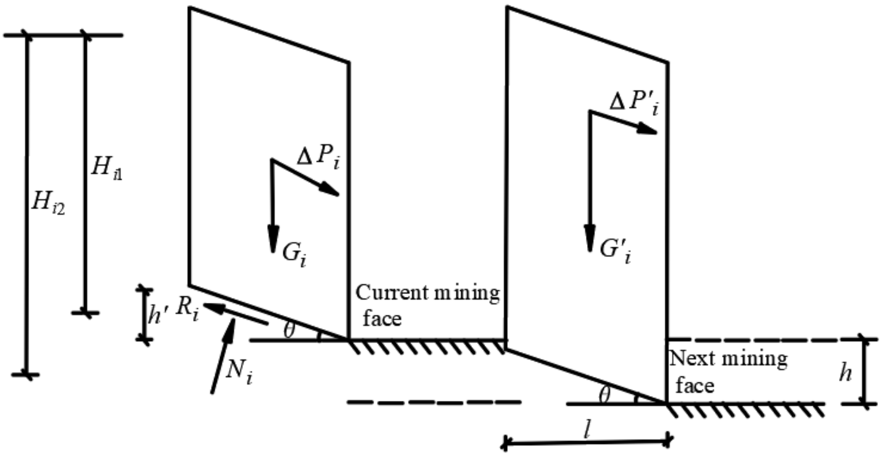

Fig 1. Force analysis of sliding strip.

Click here to access/download;Figure;Figure 2.docx

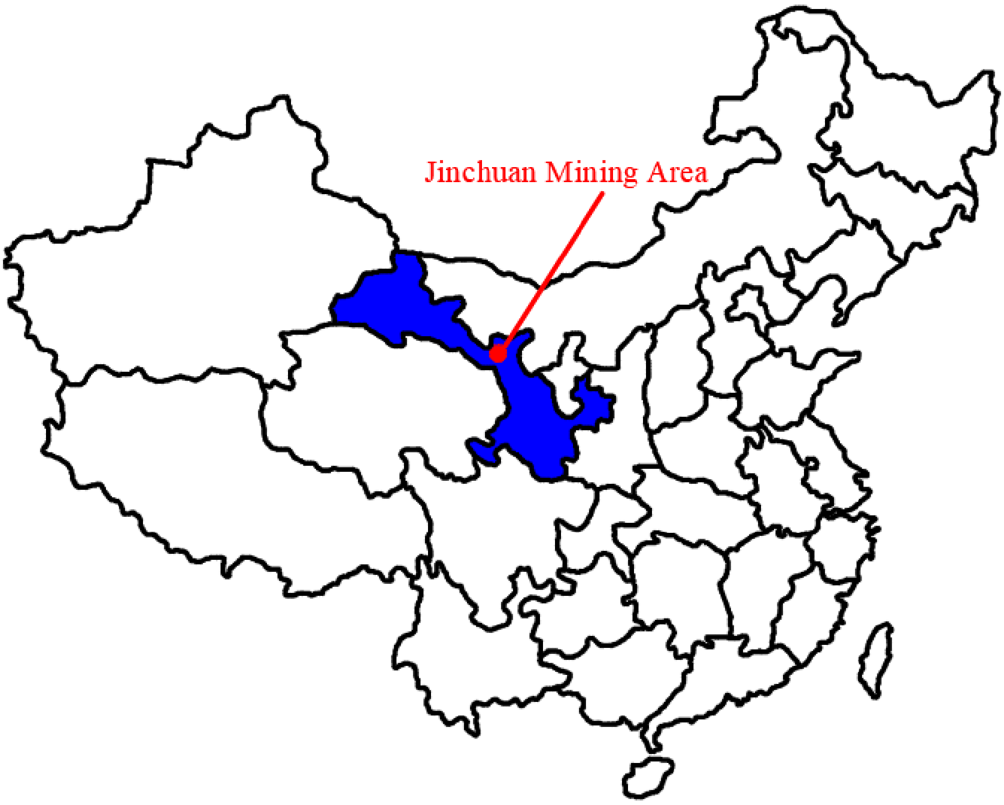

Fig 2. Location of Jinchang City in China.

Click here to access/download;Figure;Figure 3.docx

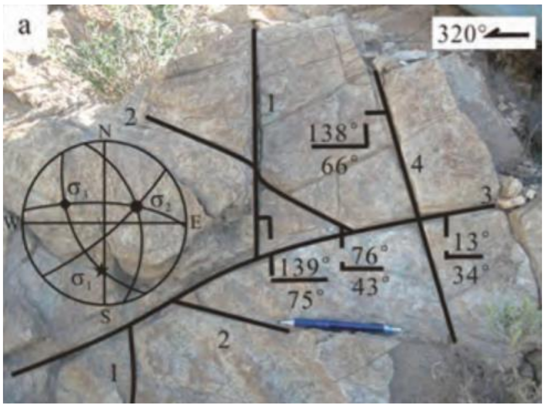

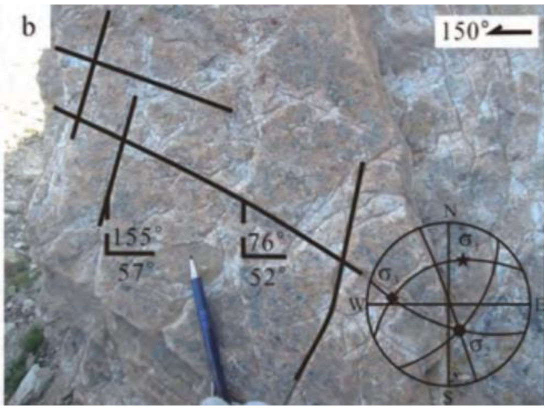

Fig 3. Fault characteristics.

Click here to access/download;Figure;Figure 4.docx

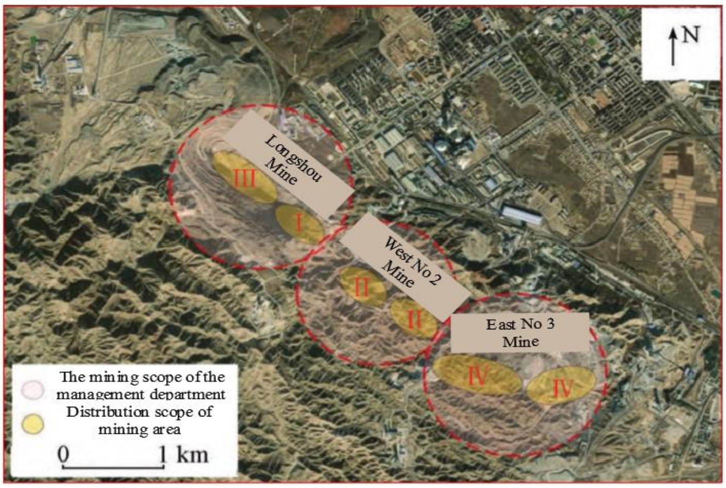

Fig 4. Mining distribution map of Jinshan Mining area.

Click here to access/download;Figure;Figure 5.docx

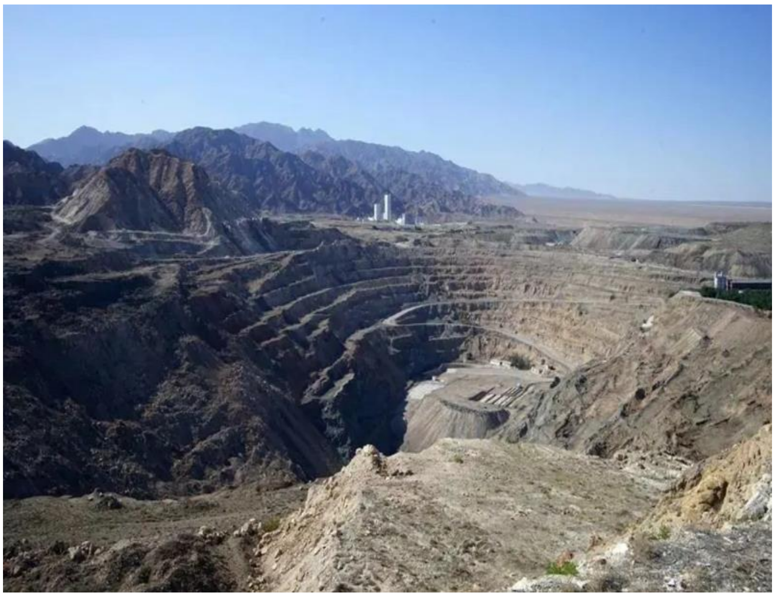

Fig 5. Multi bench slope in mining area.

Click here to access/download;Figure;Figure 6.docx

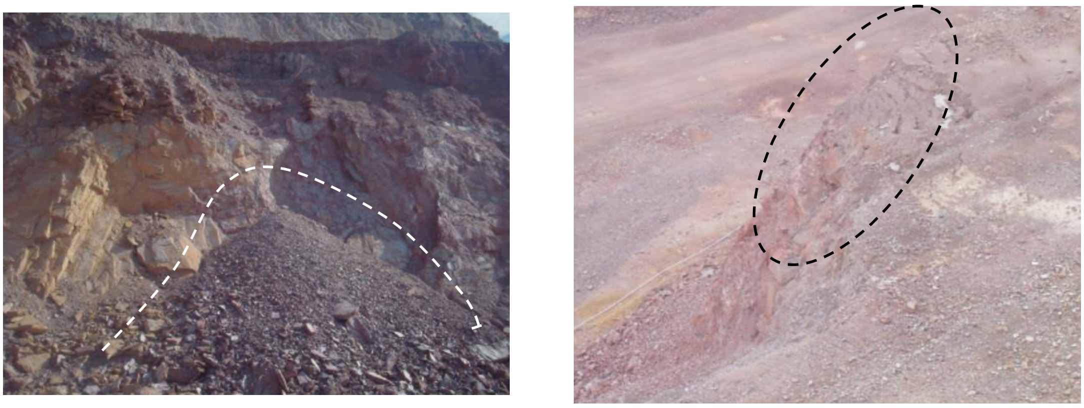

(a) Local landslide. **(b) Crack.**

Fig 6. Local failure of slope.

Click here to access/download;Figure;Figure 7.docx

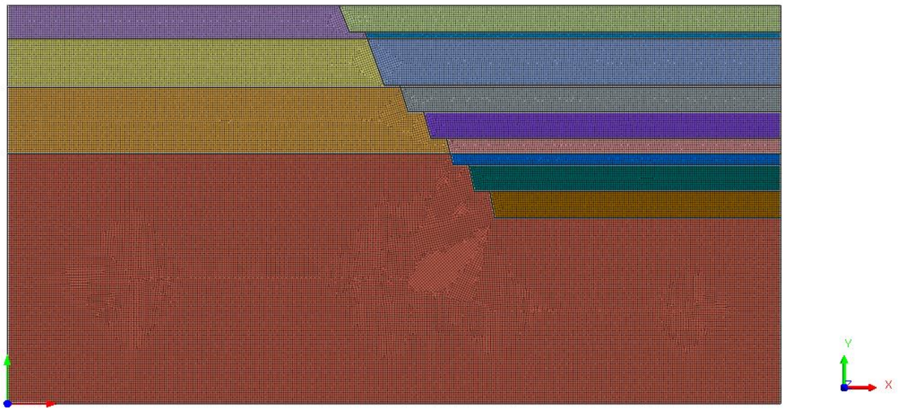

Fig 7. Excavation model of slope.

Click here to access/download;Figure;Figure 8.docx

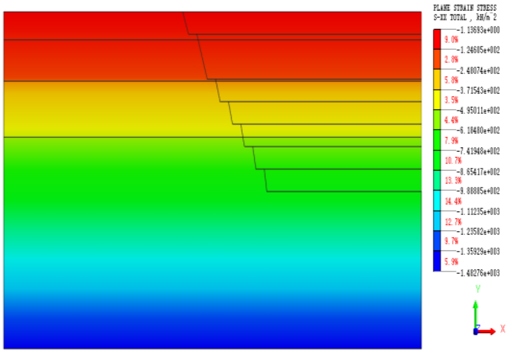

(a) Initial stress equilibrium (horizontal).

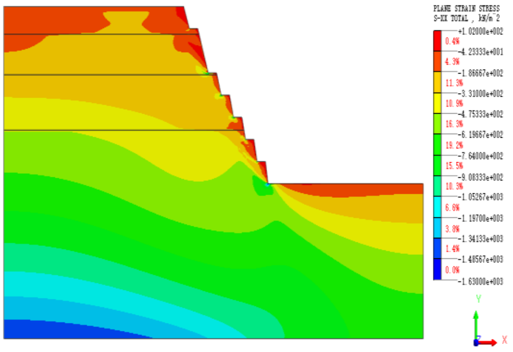

(b) Horizontal stress.

Fig 8. Horizontal stress cloud map.

Click here to access/download;Figure;Figure 9.docx

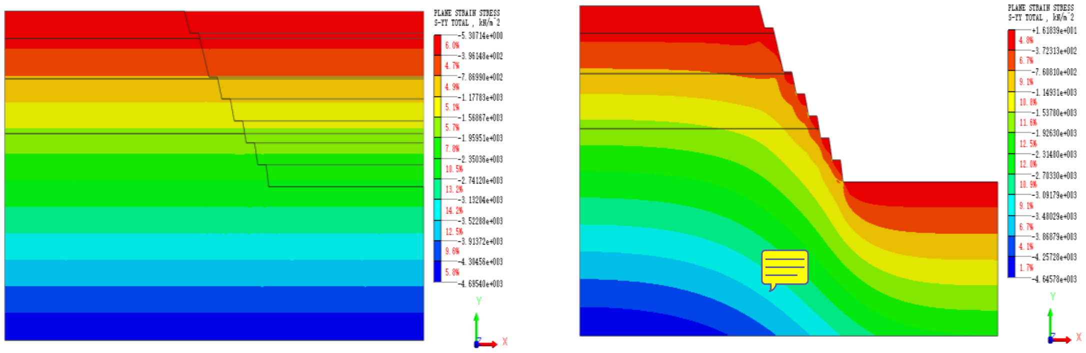

(a) Initial stress equilibrium（vertical）. **(b) Vertical stress.**

Fig 9. Vertical stress cloud map.

Click here to access/download;Figure;Figure 10.docx

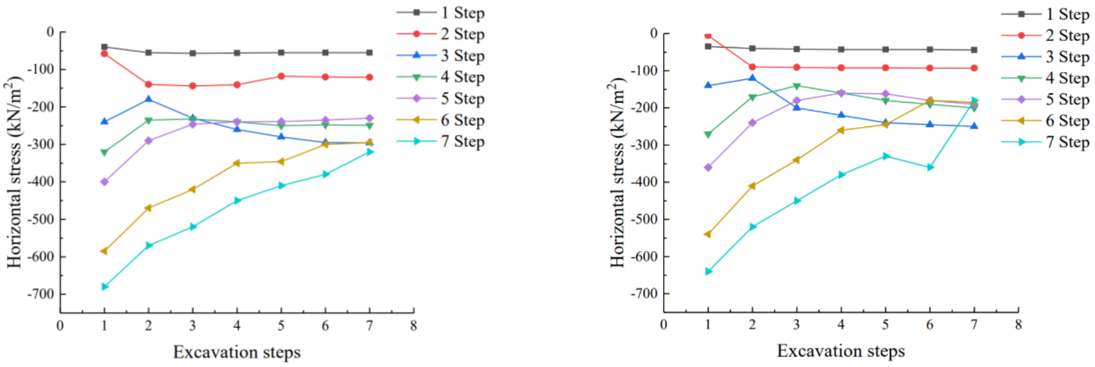

(a) In the middle of slope. **(b) At the top of the slope .**

Fig 10. The curve of horizontal stress variation of each step during slope excavation.

Click here to access/download;Figure;Figure 11.docx

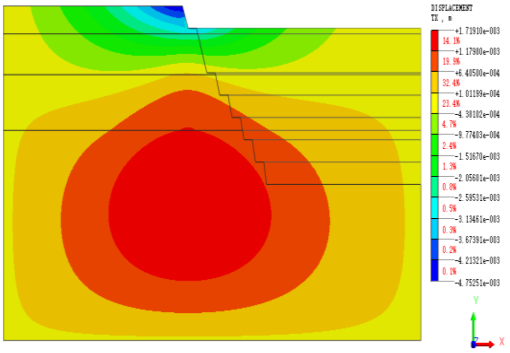

(a) **Horizontal displacement of step 1.**

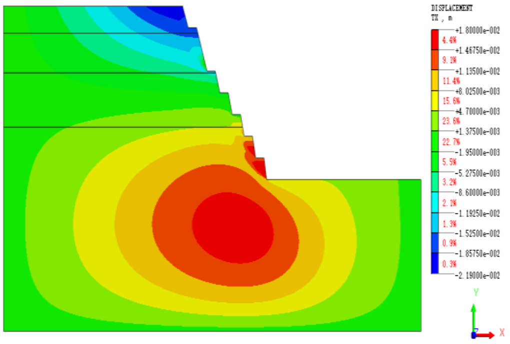

(b) **Horizontal displacement after completion of excavation.**

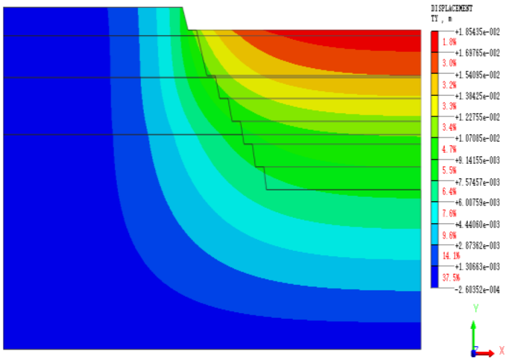

(c) Vertical displacement of step 1.

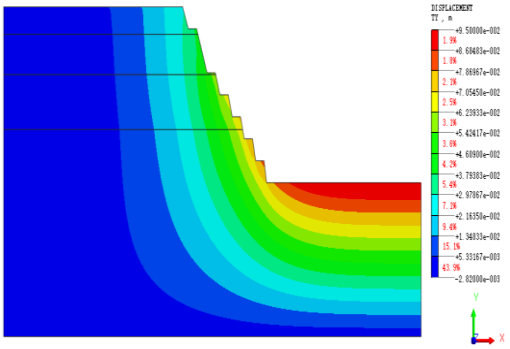

(d) Vertical displacement after excavation.

Fig 11. Horizontal and vertical displacement cloud images during slope excavation.

Click here to access/download;Figure;Figure 12.docx

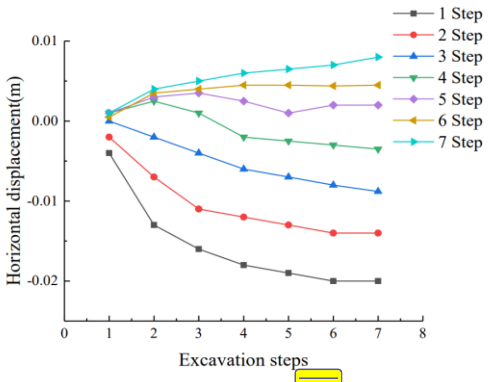

(a) Horizontal displacement.

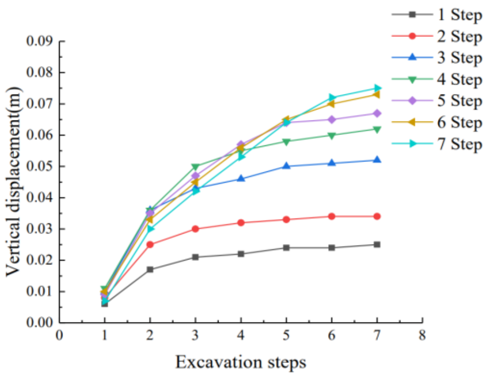

(b) The vertical displacement.

Fig 12. Displacement curves of each step during excavation.

Figure Click here to access/download;Figure;Figure 13.docx

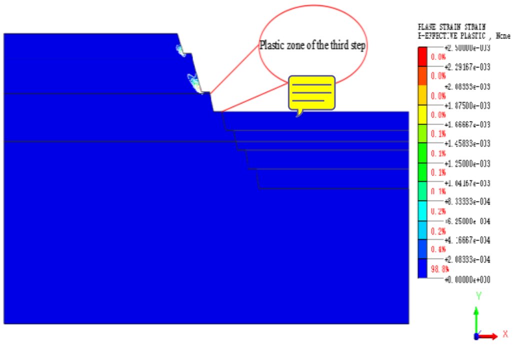

(a) Plastic zone at step 3.

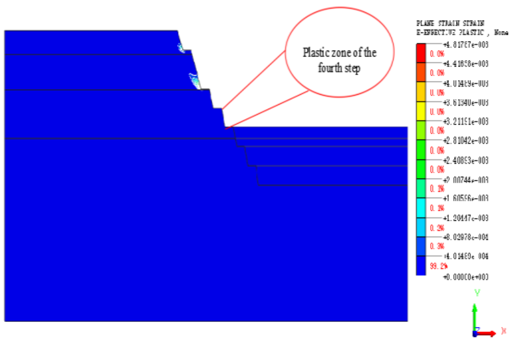

(b) Plastic zone at step 4.

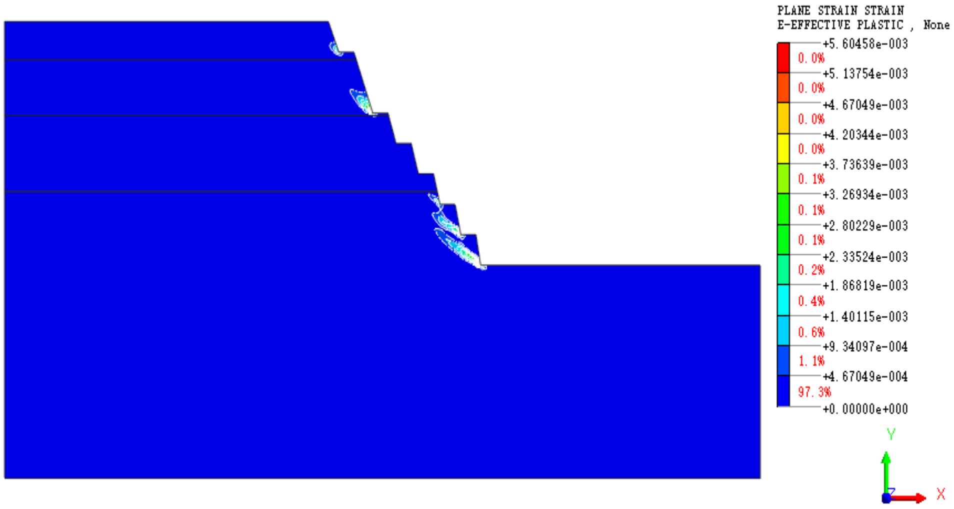

(a) Plastic zone after excavation.

Fig 13. Plastic zone after excavation.

Click here to access/download;Figure;Figure 14.docx

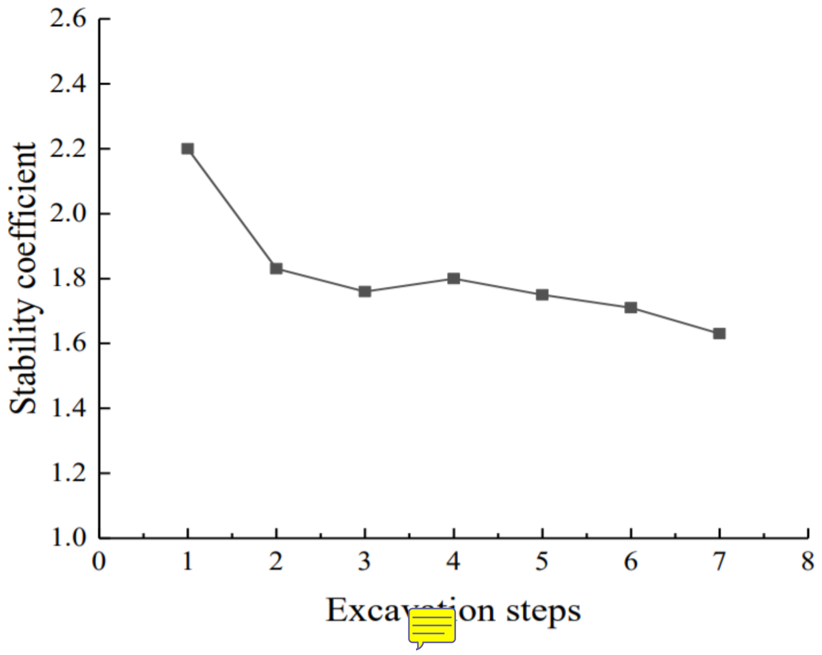

Fig 14. Variation curve of slope stability coefficient.

Click here to access/download;Figure;Figure 15.docx

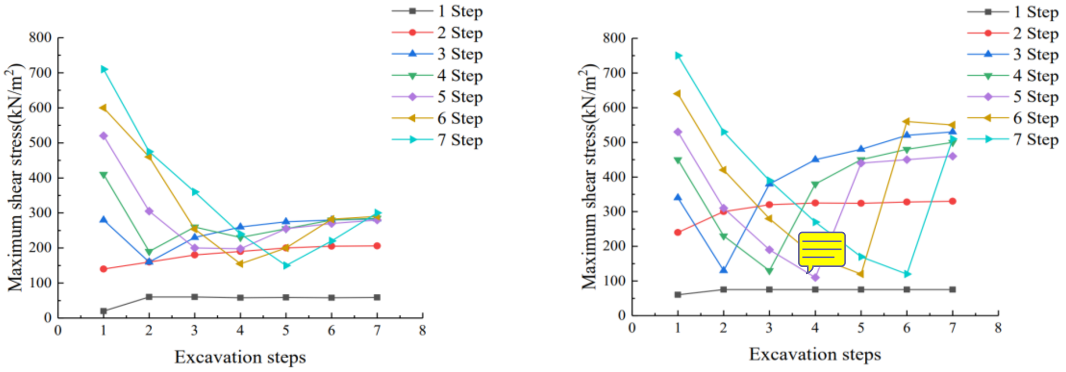

(a) In the middle of slope. **(b) Slope foot.**

Fig 15. Maximum shear stress curve of each step during slope excavation.

Click here to access/download;Figure;Figure 16.docx

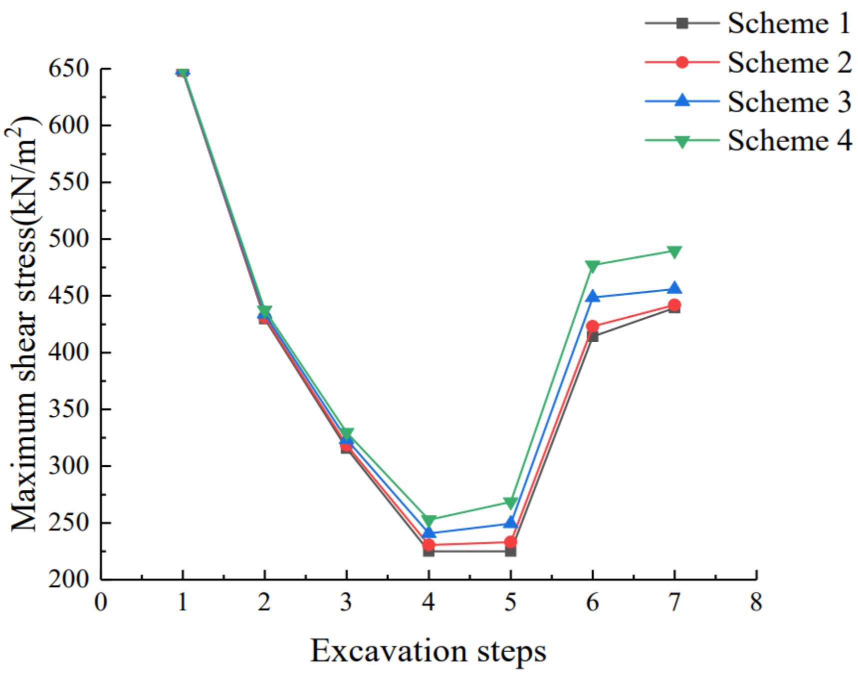

Fig 16. Curves of maximum shear stress in different experimental schemes.

Click here to access/download;Figure;Figure 17.docx

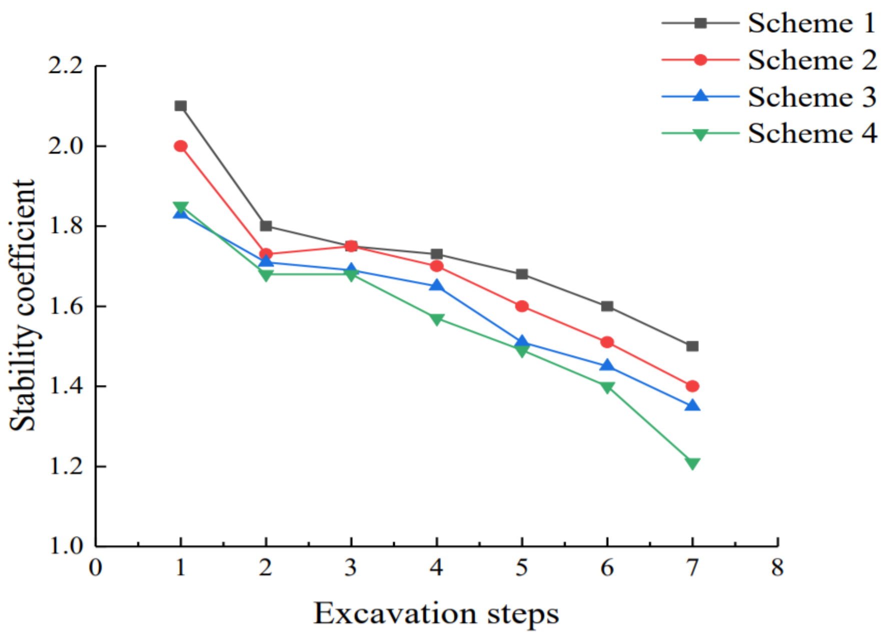

Fig 17. Curves of stability coefficients for schemes 1 to 4.
