# Supplementary material for: Analysis of stability law and optimization of slope angle during excavation of deep concave mine slope
Source: PLoS One. 2022 Jul 21;17(7):e0271700. doi: 10.1371/journal.pone.0271700 (PMC9302813; doi:10.1371/journal.pone.0271700)
Supplement: Attachment — Submitted filename: Response to Reviewers.docx [file pone.0271700.s005.docx]

Dear reviewers:

I am very grateful for your efforts and comments for the revisions of our manuscript. According to your comments and suggestions, the revisions on the manuscript ' Analysis of stability law and optimization of slope angle during excavation of deep concave mine slope' (No. PONE-D-22-08272_R1) have just been completed.

The responds to your comments and the main corrections in the manuscript are as flowing:

**(Reviewer #1:)**

1. The abstract of the paper has been redrafted, consisting the background, objective, material& method, and results with all not more than 250 words.
2. Detailed information on the characteristics of the materials have been provide in Table 1 and Table 2.
3. The tables in the paper have been checked and improved.
4. The consistency type of word has been used in paper.
5. The conclusions have been simplified and some numbers have been added to conclusions for quantitative analysis.
6. The relevant numbers have been described in conclusion 2.
7. Conclusion 3 has been quantitatively stated based on the research results.
8. The numbers in Fig 8(b) are correct. This figure is the vertical stress cloud diagram after the mine slope excavation is completed. As a result of excavation unloading, the vertical compressive stress at the foot of slope gradually decreases and tends to variety to the horizontal tensile stress. Macroscopically, the phenomenon is the rebound of excavation unloading.
9. In the process of numerical simulation, the author has considered how to reflect the time effect, but considering that the more mature time history application engineering in MIDAS-GTS software is earthquake engineering, the time history analysis in slope engineering needs to be further explored. In the future study, the author will continue to consider using time to change excavation steps, hoping to obtain some results.
10. The plasticity of material is indeed related with increasing plasticity on the model. However, the plastic zone range is very small at the third and fourth steps of excavation in Fig 13, which is due to the closure of cracks in the slope or the reduction of shear failure caused by dynamic excavation. It can also be seen from the stability coefficient that the stability of the third and fourth steps has rebounded slightly, which is more stable than the first two steps.
11. The answer to this question is similar to the answer to question 9.
12. In Fig14 (b), when the excavation of each step is completed, the shear stress at the slope toe of each step shows an increasing trend. This is because the overall height of the slope increases after the excavation of each step, and the possibility of shear failure of the slope increases, which is consistent with the law of deformation and failure of the slope.

**(Reviewer #2:)**

1.	The manuscript has undergone extensive revision for language and grammar in two aspects.

2.	The “slope Angle” has been changed to lowercase form throughout the text.

3.	The abstract has been modified, and contained the objectives, methods, findings and novelty.

4.	Previous researches on the numerical methods have been introduced in Introduction in a single chapter.

5.	Some related references on stress redistribution have been added.

6.	The text in Fig 1 has been deleted; The parameter unit has been changed from KN to kN and KPa to kPa.

7.	The advantages of Midas-GTS software and suitable comparisons between other software have been added to the article.

8.	The discussions about the optimum slope angle during the excavation with other related research have been added in the new section Discussion before Conclusion.

9.	In Reference, some references have been marked with the original language.

10.	The figures in this paper have been substantively improved.

(1) The meaning of the blue part in Fig 2 is Gansu Province.

(2) The fault is located near the platform 1732 and is part of the Jinchuan deep concave mine slope; Fig 3 has been modified.

(3) The corresponding elevation has been added in Fig 3.

(4) Scales and some lines have been added in Fig 5.

(5) The minor types of tick marks in Fig 13 and in similar figures have been deleted.

The novelty and limitation of the research have been briefed at the manuscripts.

All revisions in detail are shown in the revised manuscript with changes marked in blue. We appreciate for you warm work earnestly, and hope that the correction will meet with approval.

Once again, thank you very much for your comments and suggestions.

Sincerely yours

Lili Wu
